# Measuring oral health literacy: a scoping review of existing tools

**DOI:** 10.1186/1472-6831-14-148

**Published:** 2014-12-04

**Authors:** Virginia Dickson-Swift, Amanda Kenny, Jane Farmer, Mark Gussy, Sarah Larkins

**Affiliations:** La Trobe Rural Health School, La Trobe University, P.O. Box 199, Bendigo, Vic 3552 Australia; School of Medicine and Dentistry, James Cook University, 1 James Cook Drive, Townsville, QLD 4811 Australia

**Keywords:** Oral health literacy tools, Oral health, Dental health literacy, Health literacy, Scoping review

## Abstract

**Background:**

This article presents findings from a scoping review of tools used to measure oral health literacy. Internationally, interest in oral health literacy is driven by oral health disparities, particularly for disadvantaged groups, with conditions such as dental caries and periodontal disease contributing substantially to the global burden of disease. The increasing focus on measuring oral health literacy aligns with reasons for measuring broader health literacy, that is, by assessing oral health literacy, decisions can be made about instigating interventions at policy and practice level to improve individual and population level oral health. There are numerous tools available that measure oral health literacy using a range of indicators.

**Methods:**

A scoping review was designed to map the existing tools designed to measure oral health literacy (OHL). Key search terms were developed and mapped. Selected databases were used that identified 32 relevant studies reporting a range of OHL tools.

**Results:**

We identified 32 articles that reported a range of oral health literacy tools. Many of the studies used the Rapid Estimate of Adult Literacy in Dentistry (REALD) and/or the Test of Functional Health Literacy in Dentistry (ToFHLiD) that were developed from earlier tools designed to measure broader health literacy. These tools have been widely criticised for providing only an approximate measure of OHL based mainly on word recognition. A number of newer tools have included new measures of oral health literacy including numeracy and oral health conceptual knowledge however tools that measure important indicators of oral health literacy such as service navigation are rare.

**Conclusions:**

Findings from this scoping exercise confirm our findings from preliminary scans that the majority of tools are heavily biased towards word recognition, numeracy and reading skills, rather than what this means in terms of health behaviours and service utilisation. More recent developments have attempted to incorporate other aspects considered important, including decision making and service navigation. The incorporation of these aspects into newer tools will provide oral health researchers and policy makers with further evidence of the importance of oral health literacy when designing interventions to improve oral health.

## Background

This article presents findings from a scoping review of tools used to measure oral health literacy. Interest in this topic, as a domain of health literacy, and a determinant of health, has been growing since the late 1990s. In 2010, the United States Department of Health and Human Services released their 10-year national objectives for improving the health of all Americans [1]. In this document, the scale of oral health disparities, and the significant burden of oral disease were outlined. Oral health literacy was identified as key to promoting oral health and preventing oral health disease. Drawing on broader understandings of health literacy, oral health literacy was defined as the ‘degree to which individuals have the capacity to obtain, process and understand basic oral health information and services needed to make appropriate health decisions’.

Internationally, interest in oral health literacy is driven by oral health disparities, particularly for disadvantaged groups, with conditions such as dental caries and periodontal disease contributing substantially to the global burden of disease [2–5]. In Australia $7.5bn was spent on dental services in 2009–10, with 61% of this being direct out-of-pocket costs followed by 14% from health insurance funds. Australian Government contributions accounted for the remaining 25% [6]. The economic costs associated with poor oral health are well-documented [7, 8] and the association between oral health, and general health and wellbeing have been noted in numerous studies, with poor oral health impacting on quality of life across the lifespan [2, 7, 9]. Oral health extends beyond dental disease, with a healthy mouth central to the capacity to eat, talk and lead meaningful lives free of disease, pain or embarrassment [3, 10].

The importance of oral health beyond dental care is reflected in the WHO Global Oral Health Program, which is predicated on disease prevention and health promotion. Priority action areas of the WHO are directed at improving oral health literacy to drive increased knowledge and health-promoting behaviours [2] Authors argue that people who have poor levels of oral health literacy have poor dental health knowledge, increased dental visits and severity of oral health disease [11–13]. In the United States, the National Institute of Dental and Craniofacial Research (NIDCR) lobbied strongly for a focus on oral health literacy, arguing that poor oral health literacy is widespread and a causal factor in disparities in the oral health status between groups with high levels of oral health literacy and those without [3].

### Health literacy

Broadly, health literacy refers to skills that establish a person’s motivation and ability to access, process and use information to promote and maintain good health[14].). Increasing interest in health literacy is driven by evidence showing association between health literacy and outcomes. Low health literacy is associated with poor health knowledge, unhealthy behaviours, low usage of preventive services, poor health status, and high hospitalisation rates [15–18]. A growing body of evidence indicates that people without the health literacy skills to make sound health decisions in their everyday lives are more vulnerable and have poorer health outcomes [16, 19–21].

Early health literacy measurements focused almost exclusively on reading capacity and on links between the reading skills of adults and health outcomes. Contemporary measurements extend well beyond simply the capacity to read. Nutbeam [14, 22] conceptualises health literacy as having three distinct levels: basic/functional (reading and writing skills for everyday life); communicative/interactional (cognitive and literacy skills combined with social skills) and critical (empowerment to handle information and have control over situations). Over the last decade, researchers have extended understandings of health literacy. Nutbeam [14, 22] ,Sorensen et al. [23] with Osborne and colleagues [24] all consider writing, numeracy, speaking, listening, and understanding the healthcare system as key focal areas in any health literacy tools. The inclusion of numeracy as a key component of health literacy has been driven by claims that high percentages of the population lack the quantitative skills to understand dates and timing of medication dosages, information on appointment slips and financial information associated with healthcare [25]. In a number of studies, the ability of people, even those with good levels of reading ability, to understand numerical concepts such as probability and levels of risk has been shown to be poor [26]. It is argued that these concepts are central for promoting individual responsibility for healthcare and self-management [19, 27].

Acknowledging the limitations of previous measures of health literacy, Jordan and colleagues [28] recently developed the Health Literacy Management System (HeLMS) and Osborne and colleagues [24] the Health Literacy Questionnaire (HLQ), designed to detect a wide range of components of health literacy in community settings. Researchers have identified a myriad of reasons for measuring health literacy, varying from individual screening in clinical settings to assessing larger population level understanding and comprehension [29]. However, the central tenet is that by identifying low levels of health literacy, tailored interventions can be implemented to improve health outcomes [19, 30].

### Measuring oral health literacy

Like broader definitions of health literacy, oral health literacy refers to the capacity of a person to source, process and understand the basic information needed to make decisions about oral health. The increasing focus on measuring oral health literacy aligns with reasons for measuring broader health literacy, that is, by assessing oral health literacy, decisions can be made about instigating interventions at policy and practice level to improve individual and population level oral health [3].

The landmark 2004 United States (US) publication, *Health Literacy: A Prescription to End Confusion* [31] provides a summary of the development of oral health literacy tools, primarily from the US. The most widely used oral health literacy measurement tools are based on either the Rapid Estimate of Adult Literacy in Medicine (REALM) [32] or the Test of Functional Health Literacy in Adults (ToFHLA) [33]. The REALM is a word recognition test that evaluates participants’ ability to read from a list of medical terms and yields grade-range estimates of reading ability. ToFHLA is used to assess peoples’ literacy and numeracy skills. Findings from studies using REALM or ToFHLA indicate that adults with limited reading skills tend to know less about their disease or their treatment regimen, are less likely to engage in preventive services, and may be more limited in their ability to manage their disease [15, 32, 33].

Contemporary ways of approaching oral health literacy measurement align with broader health literacy measurement trends. Parker and Jamieson [34] include understanding the causes of poor oral health, positive oral health self-care behaviours, communication with oral health providers and ability to navigate the oral healthcare system. It is argued that people with high levels of oral health literacy know where to go for oral healthcare and how to make appointments, complete forms, comply with appointment attendance, follow-up and medication [35].

Initial tools were adapted from those used to measure general health literacy. REALM was adapted as the Rapid Estimate of Adult Literacy in Dentistry (REALD). Similarly, the Test of Functional Health Literacy in Dentistry (ToFHLiD) was developed from ToFHLA. Early tools attracted the same criticisms directed at the general health literacy versions, in that they were largely word recognition tools that did not actually measure oral health literacy *per se*, rather they provided an approximate measure of reading skills relative to oral health content [3, 36].

### The rationale for our study

As researchers interested in oral health, we were aware of the evidence of the link between oral health outcomes and oral health literacy. As part of our work, we started to source tools that could be used to measure oral health literacy in the populations that we work with. What was evident, through our initial scan of the literature, was that many of the tools were limited to measuring oral health literacy through testing word recognition, and reading skills. Drawing on the views of leaders in health literacy, we were keen to source tools that might take a broader approach to oral health literacy. Whilst it was evident that a number of tools have been developed, we failed to locate any comprehensive review of those currently available. This prompted our scoping review, and was the rationale for this study. Our aim in this article is to address this gap in knowledge by providing an overview of the current tools that have been developed internationally to measure oral health literacy.

## Methods

A scoping review was undertaken to identify what tools or instruments currently exist that measure oral health literacy across a range of different population groups. Scoping reviews are useful to map, collate and summarise existing literature on a topic and can assist researchers to identify the nature and extent of the current research evidence. Unlike systematic reviews, the focus of a scoping review is not on the assessment of the quality of the research [37] rather, the approach supports identification of a broader range of literature, including all types of study designs [38]. The work of Arksey and O’Malley [38] provides a useful methodological framework for scoping reviews. For this study, we adopted their five-stage approach: identifying the research question; identifying relevant studies; study selection; charting the data; and collating, summarising and reporting results.

### Identifying the research question

As our aim was to scope current tools designed to measure oral health literacy, we were seeking to ‘generate breadth of coverage’ [38] so a broad question and key terms were central. The question “What tools are currently available to measure oral health literacy?” guided the search strategy.

### Identifying relevant studies

Researchers have identified the need to establish clear criteria to place boundaries around a study and balance time and cost limitations with the need for a thorough review [39, 40]. In this study, key search terms were identified and a Boolean search string developed. Using truncated words and wild cards (in this case *) we aimed for a broad search that would capture all terms with the same root word. Our final string was oral* OR dental* AND (health AND literacy) AND tool or instrument.

An initial search of Google Scholar was carried out to determine the likely size and relevance of the key terms, but the results were not included in our findings due to the lack of replicability from this search engine [41]. To determine an appropriate time frame for the review, the Google Scholar search located minimal research on oral health literacy tools prior to 2007 so this date was chosen as appropriate for this study. Databases searched included CINAHL, ProQuest, Informit, Pub Med and Medline. International studies designed for specific cultural groups were included to provide a comprehensive overview of the tools utilised for diverse samples. A search of the Cochrane Library located one registered trial, describing an oral health literacy intervention protocol for Indigenous adults in an Australian rural setting [42]. Inclusion and exclusion criteria, consistent with our review purpose, were developed and are outlined in Table 1.

**Table 1:** **Inclusion and exclusion criteria**

Criterion	Inclusion	Exclusion
Time period	January 2007 to September 2014	Any study outside these dates
Language	English	Non-English
Type of article	Original research article published in a peer reviewed journal that provides a description of a tool. The article may then proceed to use the tool or report of instrument or tool	Any publication that was not original research, peer-reviewed journal article and/or unpublished. For example, PhD theses and reports were excluded. Articles that do not report using a tool or instrument were also excluded.
Study focus	Oral or dental health literacy measurement tools or instruments	No reference to oral health literacy tools or instruments
Geographical area of interest	International studies including those with specific cultural groups	Nil
Setting	Any	Nil

### Study selection

Using the developed search terms, 239 articles were identified that used various oral health literacy tools. After deletion of duplicates, 123 articles remained. The bibliographic software program Endnote X6 was used to import and manage references. The title, abstract and keywords of the articles were scrutinised against the inclusion and exclusion criteria with research team members agreeing and confirming the elimination of irrelevant studies. Through this process, 32 articles were included in the final review.

### Data charting and collation

Taking the included studies, and consistent with the fourth and fifth stages of Arksey and O’Malley’s framework, a chronological overview of the current tools used to measure oral health literacy was developed (see Table 2). Using an Excel spreadsheet, the studies were charted and summaries developed that included author, journal, publication year, research question or aim, setting, sample, and tool/instrument used (see Table 3).

**Table 2:** **Chronological overview of oral health literacy tools**

Abbreviation	Name of tool	Year	Authors	Type of tool
REALD-99	Rapid Estimate of Adult Literacy in Dentistry	2007	Richman et al.	99 item word recognition
REALD-30	Rapid Estimate of Adult Literacy in Dentistry -30	2007	Lee et al.	30 item word recognition common dental words
ToFHLiD	Test of Functional Health Literacy in Dentistry	2007	Gong et al.	Reading comprehension and numeracy 68 item reading comprehension and 12 item numeracy
OHLI	Oral Health Literacy Instrument	2009	Sabbahi et al.	Reading comprehension and numeracy
REALM-D	Rapid Estimate of Adult Literacy in Medicine and Dentistry	2010	Atchinson et al.	84 item word recognition
CMOHK	Comprehensive Measure of Oral Health Knowledge	2010	Macek et al.	44 questions conceptual knowledge
BHLOHKP	Baltimore Health Literacy and Oral Health Knowledge Project survey	2011	Macek et al.	44 item questionnaire conceptual knowledge across 4 domains
HKREALD-30	Hong Kong Rapid Estimate of Adult Literacy in Dentistry	2012	Wong et al.	Adaptation of the REALD-99 translated to Chinese and shortened to the REALD-30
		2013	Bridges et al.	
OHLA-S	Oral Health Literacy Assessment-Spanish	2012	Lee et al.	Developed using the REALD-30 word recognition and comprehension
OHLA-E	Oral Health Literacy Assessment-English	2012	Lee et al.	Developed using the REALD-30 word recognition and comprehension
REALMD-20	Rapid Estimate of Adult Literacy in Dentistry-20	2013	Gironda et al.	20 item word recognition
HKOHLAT-P	Hong Kong Oral Health Literacy Assessment Task for Paediatric Dentistry	2013	Wong et al.	Mainly literacy and numeracy tasks
		2013	Bridges et al.	
OHL-AQ	Oral Health Literacy Adults Questionnaire	2013	Sistani et al.	17 items in 4 sections, reading comprehension, numeracy, literacy and decision making
HeLD	Health Literacy in Dentistry	2013	Jones et al.	Modelled on the HeLMS

**Table 3:** **Overview of studies using oral health literacy tools 2007-2013**

Authors	Year	Title	Journal	Aim	Sample	n=	Setting	Tool used
Gong, D., Lee, J., Rozier, G., Pahel, B., Richman, J., Vann, W.	2007	Development and testing of the Test of Functional Health Literacy in Dentistry (ToFHLiD)	Journal of Public Health Dentistry	To evaluate the reliability and validity of the ToFHLiD	Parents of paediatric patients	102	Caregivers of paediatric dental patients seeking care in two dental clinics in North Carolina	ToFHLiD
Jones, M., Lee, J., Rozier, G.	2007	Oral health literacy among adult patients seeking dental care	Journal of the American Dental Association	To examine the association of knowledge, dental care visits and oral health status with oral health literacy in dental patients	Adult patients	101	Convenience sample of adult patients presenting for treatment at private dental practices in North Carolina	REALD-30 and short interview
Lee, J., Rozier, G., Lee, S., Bender, D., Ruiz, R.	2007	Development of a word recognition instrument to test health literacy in dentistry: The REALD-30- A brief communication	Journal of Public Health Dentistry	To develop and pilot test a dental word recognition instrument	Adult patients	200	Ambulatory Care Centre at the University of North Carolina Hospital	REALD-30 and interview that included the TOFHLA & REALM & OHIP-14
Richman, K., Lee, J., Rozier, G., Gong, D., Pahel, B., Vann, W.	2007	Evaluation of a word recognition instrument to test health literacy in dentistry: The REALD-99	American Association of Public Health Dentistry	To evaluate a dental health literacy word recognition instrument	Parents of paediatric patients	102	Parents and caregivers of paediatric dental patients from the UNC-CH School of Dentistry Paediatric Dental Clinics and from Orange County Dental Clinics	REALD-99
Jackson, R., Eckert, G.	2008	Health literacy in an adult dental research population: A pilot study	American Association of Public Health Dentistry	To gather data concerning the level of health literacy in adults who frequently volunteer for clinical research programs	Adults enrolled in the Oral Health Research Institute School of Dentistry	100	Oral Health Research Institute of Indiana University School of Dentistry	S-ToFHLA
Sabbahi.D., Lawrence, H., Limeback, H., Rootman, I.	2009	Development and evaluation of an oral health literacy instrument for adults	Community Dentistry and Oral Epidemiology	To develop and validate an instrument to measure functional oral health literacy of adults	Adult patients	100	Convenience sample of patients attending the Faculty of Dentistry clinics at the University of Toronto	Oral Health Knowledge test, OHLI, ToHFLA
Atchison, K., Gironda, M., Messadi, D., Der-Martirosian, C.	2010	Screening for oral health literacy in an urban dental clinic	Journal of Public Health Dentistry	To evaluate a health literacy instrument based on the REALM that incorporates dental and medical terms into one 84-item REALM-D measure and determine its association with patient characteristics of a culturally diverse dental clinic population	Adult patients	200	Oral health clinic urban centre Los Angeles, California	REALM-D
Macek, M., Haynes, D., Wells, W., Bauer-Leffler, S., Cotten, P., Parker, R.	2010	Measuring conceptual health knowledge in the context of oral health literacy: preliminary results	Journal of Public Health Dentistry	To assess the validity and reliability of a new instrument and describe conceptual oral health knowledge among a sample of low-income adults	Adult residents of Baltimore	100	Baltimore residents randomly selected from a list of those that had landlines	REALM and the S-ToFHLA to develop CMOHK
Parker, E., Jamieson, L.	2010	Associations between Indigenous Australian oral health literacy and self-reported oral health outcomes	BMC Oral Health	To determine oral health literacy (REALD-30) and oral health literacy-related outcome associations, and to calculate if oral health literacy-related outcomes are risk indicators for poor self-reported oral health among rural-dwelling Indigenous Australians	Indigenous adults	468	Convenience sample of Indigenous adults living in the Port Augusta region of Australia	REALD-30 and measures from OHL-14
Vann, W., Lee, J., Baker, D., Divaris, K.	2010	Oral health literacy among female caregivers: Impact on oral health outcomes in early childhood	Journal of Dental Research	To investigate the association of female caregivers’ oral health literacy with their knowledge, behaviours and the reported oral health status of their young children	Child/ caregiver dyads from the Carolina Oral Health Literacy Project	1273	Caregivers and children enrolled in the Women’s Infants and Children’s (WIC)Supplemental Nutrition Program in North Carolina	REALD-30 and oral hygiene behaviours
Wells, P., Caplan, D., Strauss, R., Bell, D., George, M.	2010	An oral health survey of the Lumbee tribe in South-eastern North Carolina	The Journal of Dental Hygiene	To evaluate access to dental care issues, oral health knowledge and oral health-related quality of life of the Lumbee tribe	Adult Lumbee tribe members	118	Convenience sample of American Indian attending the Lumbee Tribe Homecoming in North Carolina	OHIP-14 and survey
Lee, Y., Divaris, K., Baker, A. Vann, W.	2011	The relationship of oral health literacy with oral health-related quality of life in a multi-racial sample of low-income female caregivers	Health and Quality of Life Outcomes	To investigate the association between oral health literacy (OHL) and Oral-Health Related Quality of Life (OHRQoL) and explore the racial differences therein among a low-income community-based group of female WIC participants.	Low income adult females enrolled in WIC program	1405	Community setting of adult women enrolled in the Women, Infants and Children’s(WIC) Supplemental Nutrition Program in 7 counties in North Carolina.	OHIP-14, REALD-30
Lee, J., Divaris, K., Baker, A., Rozier. R., Lee, S., Vann, W.	2011	Oral health literacy levels among a low-income WIC population	Journal of Public Health Dentistry	To determine oral health literacy (OHL) levels and explore potential racial differences in a low-income population	Care givers of paediatric patients	1405	Community setting of adult women enrolled in the Women, Infants and Children’s(WIC) Supplemental Nutrition Program in 7 counties in North Carolina.	REALD-30 and survey
Macek, M., Manski, M., Schneiderman, T., Meakin, S., Haynes, D., Wells, W., Bauer-Leffler, S., Cotton, A. Parker, R.	2011	Knowledge of oral health issues among low-income Baltimore adult: A pilot study	Journal of Dental Hygiene	Pilot study to document conceptual knowledge of oral health among low income adults in Baltimore	Low income adults in Baltimore	100	Baltimore residents randomly selected from a list of those that had landlines	BHLOHKP
Stucky, B., Lee, J., Lee, S., Rozier, R.,	2011	Development of the two-stage Rapid Estimate of Adult Literacy in Dentistry	Community Dentistry and Oral Epidemiology	To revise the 30 item Rapid Estimate of Adult Literacy in Dentistry (REALD-30) into a more efficient and easier-to-use two-stage model	Low income adults (primarily women) enrolled in the WIC program	1405	Low income adults (primarily women) enrolled in the North Carolina WIC supplemental nutrition program	REALD-30, TS-REALD
Lee, J., Divaris, K., Baker, A., Rozier, R., Vann, W.	2012	The relationship of oral health literacy and self-efficacy with oral health status and dental neglect	American Journal of Public Health	To examine the association of oral health literacy (OHL) with oral health status (OHS) and dental neglect (DN) and whether self efficacy mediated or modified these associations	Female caregivers	1280	Community setting of adult women enrolled in the Women, Infants and Children’s WIC Supplemental Nutrition Program (in 7 counties in North Carolina.	REALD-30
Lee, J., Stucky, B., Rozier, G., Lee, S., Zeldin, L.	2012	Oral health literacy assessment: development of an oral health literacy instrument for Spanish speakers	Journal of Public Health Dentistry	To develop an oral health literacy instrument for Spanish-speaking adults , evaluate its psychometric properties and determine its comparability to the English version	Adults fluent in English or Spanish at various sites in North Carolina	405	Sites that included WIC clinics in various regions of North Carolina, Early Head Start Centre and a continuity care clinic in North Carolina	OHLA, OHLA-S
Parker, E., Misan, G., Chong, A., Mills, A., Roberts-Thomson, K., Horowitz, A., Jamieson, L.	2012	An oral health literacy intervention for Indigenous adults living in a rural setting in Australia	BMC Public Health	To determine if implementation of a functional, context-specific oral health literacy intervention improves oral health literacy-related outcomes measured by use of dental services, and assessment of oral health knowledge, oral health self-care and oral health-related self-efficacy	Indigenous adults	400	RCT with randomisation	Adaptation of the HeLM
Wehmeyer, M., Corwin, C., Guthmiller, J., Lee, J.,	2012	The impact of oral health literacy on periodontal health status	American Association of Public Health Dentistry	To describe the oral health literacy (OHL) among periodontal patients and to examine its association with periodontal health status	Adult patients	121	Convenience sample of adult patients presenting for initial consultation appointment to the University of North Carolina Graduate Periodontology Clinic	REALD-30 and survey and periodontal exam
Wong, H., Bridges, S., You. C., McGrath, C., Au, T., Parthasarathy, D.	2012	Development and validation of Hong Kong Rapid Estimate of Adult Literacy in Dentistry	Journal of Investigative and Clinical Dentistry	To develop and validate an instrument , the Hong Kong Rapid Estimate of Adult Literacy in Dentistry	Parents of paediatric dental patients	200	Convenience sample of parents of paediatric patients attending the Paediatric Dentistry Clinic in Hong Kong	REALD-99 translated to Chinese and modified to the HKREALD-30 and clinical examination
Bridges, S., Parthasarathy, D., Au, T., Wong, H., Yiu, C., McGrath, C.	2013	Development of functional oral health literacy assessment instruments: Application of literacy and cognitive theories	Journal of Public Health Dentistry	Development of a new literacy assessment instrument to establish content and face validity.	care givers of paediatric patients	Not specified	Various clinics and community settings in Hong Kong	HKOHLAT-P
Bridges, S., Parthasarathy, D., Wong, H., Yiu, C., Au, T., McGrath, C.	2013	The relationship between caregiver functional oral health literacy and child oral health status	Patient Education and Counselling	To describe the relationship between caregiver’s oral health literacy (OHL) and the oral health status of their children in an Asian population	care givers of paediatric patients	301	Child/caregiver dyads from kindergarten in Hong Kong	HKREALD-30 and HKOHLAT-P
Gironda, Der-Martirosian, C., Messadi, D., Holtzman, J. Atchinson, K.	2013	A brief 20-item dental/medical health literacy screen (REALMD-20)	Journal of Public Health Dentistry	To introduce a brief 20 item screener for limited dental/medical health literacy among adult dental patients	Adult patients seeking treatment for the first time	200	Patients seeking treatment for the first time at an Oral Diagnosis Clinic at a School of Dentistry in the US.	REALMD-20
Hom, J., Lee, J., Divaris, K., Baker, A., Vann, W.	2013	Oral health literacy and knowledge among patients who are pregnant for the first time	The Journal of the American Dental Association	To determine the levels of and examine the associations of oral health literacy (OHL) and oral health knowledge in low income patients who were pregnant for the first time	Low income women pregnant for the first time	119	Subset of women pregnant for the first time in WIC project in North Carolina	REALD-30 and OHL survey
Jamieson, L., Divaris, K., Parker, E., Lee, J.	2013	Oral health literacy comparisons between Indigenous Australians and American Indians	Community Dental Health	To compare oral health literacy (OHL) levels between two profoundly disadvantaged groups, Indigenous Australians and American Indians and to explore the differences in socio-demographic, dental service utilisation, self-reported oral health indicators, and oral health-related quality of life correlates of OHL among the above.	Indigenous adults (Australia), American Indians (North Carolina)	468 (Aus) 254 (USA)	Convenience sample of Indigenous adults living in the Port Augusta region of Australia and a convenience sample of caregivers attending the WIC clinics at selected sites in North Carolina	REALD-30 and OHP-14
Jones, K., Parker, E., Mills, H., Horowitz, A., Brennan, D., Jamieson, L.	2013	Development and psychometric validation of a Health Literacy in Dentistry scale (HeLD)	Community Dental Health	To develop and validate a culturally-appropriate Health Literacy in Dentistry (HeLD) instrument for use amongst Indigenous Australians	Indigenous adults	209	Convenience sample of Indigenous adults living in the Port Augusta region of Australia	HeLD
Sistani, M., Montazeri, A., Yazdani, R., Murtomaa, H.	2013	New oral health literacy instrument for public health: development and pilot testing	Journal of Investigative and Clinical Dentistry	To develop a functional oral health literacy (OHL) instrument for adults including new measures of literacy skills (OHL-AQ)	Adult citizens living in Tehran	97	Randomly selected households in Tehran	OHL-AQ
Sistani, M., Yazdani, R. Virtanen, J., Pakdaman,A., Murtomaa, H.	2013	Determinants of oral health: Does oral health literacy matter?	ISRN Dentistry	To evaluate oral health literacy, independent of other oral health determinants , as a risk indicator for self-reported oral health	Adults living in Tehran	1031	Random area sampling in Tehran	OHL-AQ
Sistani, M., Yazdani, R. Virtanen, J., Pakdaman,A., Murtomaa, H.	2013	Oral health literacy and information sources among adults in Tehran, Iran	Community Dental Health	To assess oral health literacy level and oral health information of Iranian adults in Tehran, and to determine the factors related to oral health literacy	Adults	1031	Multi-stage random sample from Tehran, Iran	OHL-AQ
Ueno, M. Takeuchi, S., Oshiro, A., Kawaguchi, Y.	2013	Relationship between oral health literacy and oral health behaviors and clinical status in Japanese adults	Journal of Dental Sciences	To investigate how oral health literacy relates to oral health behaviors, as well as clinical dental and periodontal conditions.	Adult residents of Akita in Japan	518	Adult residents aged older than 20 living in Akita, Japan	Self administered questionnaire and dental exam
Wong, H., Bridges, S., Yiu, C., McGrath, C., Au, T., Parthasarathy, D.,	2013	Validation of the Hong Kong Literacy Assessment Task for Paediatric Dentistry (HKOHLAT-P)	International Journal of Paediatric Dentistry	To validate an original instrument. The Hong Kong Oral Health Literacy Assessment Task (HKOHLAT-P) for paediatric dentistry	Parent/ Child dyads	200 pairs	Convenience sample of 200 pairs of parents/children attending the Paediatric Dentistry Clinic in Hong Kong	HKOHLAT-P, ToFHLiD, ECO-HIS and interviews using HKREALD-30
Holtzman, J., Atchison, J., Gironda, M., Radbod, R., Gornbein, J.	2013	The association between oral health literacy and failed appointments in adults attending a university-based general dental clinic	Community Dentistry and Oral Epidemiology	To determine the association between personal characteristics, a person’s oral health literacy and failing to show for dental appointments	Adults	200	Secondary analysis of 200 adult patients at a university dental clinic	REALM-D and socio-demographic survey

### Findings

The following tables summarise tools identified through the scoping review.

## Results and discussion

As outlined in Tables 2 and 3, the scoping review identified several oral health literacy tools that have been used since 2007. The most frequently used are those based on the Rapid Estimate of Adult Literacy in Medicine (REALM) developed by Davis and colleagues in 1993 [32]. Adaptations to measure oral health literacy include REALD 99 [43], REALD-30 [44–46], REALM-D [46, 48], and most recently, REALMD-20 [48]. The REALD tools are essentially word recognition tests that consist of dental terms from the American Dental Association Glossary of Common Dental Terminology and patient education materials [44–48]. The tools have reportedly shown to be valid and reliable in measuring word recognition. Adding 69 new words to REALD-30, thereby creating REALD-99, did not improve results sufficiently to justify extending the list of dental words [43]. Gironda and colleagues [49] developed a shortened version, REALMD-20, for clinicians to detect limited medical/dental health literacy in patients attending for treatment in dental/medical clinics. The authors acknowledge that the tool is useful for measuring the reading ability of patients and whilst not an effective measure of comprehensive health literacy, it does provide clinicians with a useful tool to use as a screening instrument.

The other popular oral health literacy tool identified in the review is based on the Test of Functional Health Literacy in Adults (ToFHLA) [33], a widely used measure of health literacy. This instrument, the Test of Functional Health Literacy in Dentistry (ToFHLiD), was developed by Gong and colleagues [50] and consists of a 68-item reading comprehension section and a 12-item numeracy section. Initial validation of ToFHLiD showed a low internal reliability but a strong convergent validity since the ToFHLiD scores were highly correlated to the REALD-99 scores. In addition, ToFHLiD showed a moderate ability to discriminate between dental and medical literacy. Despite these limitations it is often used in conjunction with other tools designed to measure oral health literacy levels (see Table 3).

In more recent years a variety of other oral health literacy measurements tools have been developed. The Oral Health Literacy Instrument (OHLI) was developed by Sabbahi et al. [11] for use with adult dental patients. This tool (like the ToFHLA) contains both reading comprehension and numeracy sections. The reading comprehension is a 38 item test with words omitted from one passage on dental caries and another on periodontal disease. The numeracy section has 19 items to test comprehension of directions for taking common prescriptions associated with dental treatment, post extraction instructions and dental appointments. Sabahhi and colleagues added an oral health knowledge test to the tool that was designed to evaluate the patients’ general dental knowledge to be used as a predictor of functional health literacy. The knowledge test consists of seven pictures depicting 17 labelled items such as perioral and intra-oral structures, oral diseases and conditions, dental fillings, a dental prosthesis, and different oral hygiene aids. To complete this test patients were asked to match the pictures to the words. Used with a sample of 100 patients it was shown to be a valid and reliable instrument when compared to other OHL tools. The authors acknowledge that the OHLI measures the patient’s ability to perform oral health literacy-related tasks that require reading, comprehension and numeracy skills and whilst it provides a useful estimate of these abilities it does not capture the full complement of literacy skills. The authors conclude that more work is needed to investigate the instrument’s predictive validity and sensitivity to change using oral health outcomes with population groups known to be at high risk of low functional oral health literacy [11].

In 2010, Macek [51] and colleagues used a combination of the REALM, the Short Test of Functional Health Literacy in Adults (Short-TOFHLA) and later a new survey they developed to explore conceptual oral health knowledge [52]. These were administered to 100 adults in Baltimore. The respondents were also asked about socio-demographics, dental health, and utilization of dental services. Psychometric analysis was used to identify a subset of oral health knowledge questions from the new survey instrument. The resulting Comprehensive Measure of Oral Health Knowledge (CMOHK) was categorized into three levels of knowledge (poor, fair, good). This preliminary study yielded a new measure of oral health conceptual knowledge, available for use in future oral health literacy studies.

Similarly many researchers interested in exploring oral health literacy with low income populations have utlised the REALD-30 and oral hygiene behaviours to investigate the association of female caregivers’ oral health literacy with their knowledge, behaviours and the reported oral health status of their young children [13, 45, 51–56] . The sample for these studies were drawn exclusively from those enrolled in the Women’s Infants and Children’s (WIC) supplemental nutrition program in North Carolina.

Few tools had been adapted for specific populations or cultural groupings. The Hong Kong Rapid Estimate of Adult Literacy in Dentistry (HKREALD-30) [57, 58], the Hong Kong Oral Health Literacy Assessment Task for Paediatric Dentistry (HKOHLAT-P) [56, 58–60], and the Oral Health Literacy Assessment-Spanish (OHLA-S) [54] are exceptions. Other tools have been adapted for use with specific cultural groups [34, 54–66]. A recent study by Parker and Jamieson [34] used REALD-30 and oral health literacy-related outcome associations to calculate risk indicators for poor self-reported oral health among rural-dwelling Indigenous Australians. This study aimed to determine the relationship between oral health literacy, as assessed by REALD-30 and oral health literacy-related outcomes. The researchers identified individuals’ oral health knowledge, oral health self-care and utilisation of dental services, to determine if these factors (often measured in existing tests of oral health literacy) are risk indicators for seven domains of poor self-reported oral health. Parker and Jamieson acknowledge the shortcomings of REALD-30 particularly that it measures word recognition only, with no test of comprehension or functional oral health literacy. The authors initially included questions from the TOFHLiD, as an attempt to measure broader aspects of oral health literacy, including reading comprehension and numerical ability but these were removed after trialling with the Indigenous reference group, members of which identified a potential lack of acceptance within their community. The researchers note that some participants felt uncomfortable with the instrument, feeling like they were being “tested” and “judged” [34]. The findings of this study confirmed that those with poorer oral health literacy, as measured by REALD-30, had poorer oral health knowledge and engaged in more harmful oral health behaviours.

Seeking to develop a reliable, valid and culturally appropriate instrument to assess oral health literacy among vulnerable groups, Jones et al developed the Health Literacy in Dentistry scale (HeLD). Using the Health Literacy Measurement Scale (HeLMS) as a foundation, a number of theoretical constructs were included which assume “a person’s ability to seek, understand and use oral health information is important in being able to access and benefit from oral health care services” [63]. The HeLD has eight domains which mirror those used in the HeLMS. The HeLD accounts for the multidimensional nature of oral health literacy and encompasses the domains of communication, access, receptivity, understanding, utilisation, support and economic barriers which have all been shown to impact on oral health status. The results of a HeLD pilot with 209 Indigenous adults highlight the potential for using the instrument across a variety of health care settings whilst “still allowing reliable international comparisons to be made” [63:6]. The researchers state that results of studies utilising this tool will be of interest to all those working on OHL measurement with both marginalised and mainstream groups [63].

Sistani and colleagues [64–66] developed and pilot tested an Oral Health Literacy Adults Questionnaire (OHL-AQ) which they state is valid and reliable. The OHL-AQ comprises four sections: reading comprehension, numeracy, listening, and decision-making. This tool was developed to address limitations of existing oral health literacy instruments, including their length, lack of generalizability across populations, and their focus on measuring either the ability of a person to read specific dental health vocabularies or the ability to read and comprehend oral health information and calculate numbers. Their aim was to develop a generic oral health literacy instrument for adults that included measures of listening and appropriate decision making. They argue that the OHL-AQ is a valid and reliable instrument for the functional assessment of adults’ oral health literacy in community or population-based studies and because it is short and easy to use, could be used in clinical or research settings to improve oral health-related literacy skills and dentist–patient communication. The authors conclude that adding two new measures (listening and decision-making) improves the performance and quality of the existing instruments. They highlight that future research should include a larger population, in order to demonstrate the determinants of oral health literacy, particularly amongst those with limited general literacy skills.

## Conclusions

The most widely used oral health literacy measurement tools are based on either the REALM or the TOFHLA. Findings from this scoping exercise confirm our findings from preliminary scans that the majority of tools are heavily biased towards word recognition, numeracy and reading skills, rather than what this means in terms of health behaviours and service utilisation. More recent developments have attempted to incorporate other aspects considered important, including decision making and possibly service navigation. The incorporation of these aspects should increase the validity of these tools as a measure of oral health literacy in its broader sense incorporating communicative/interactional and critical levels however formal validation work is required. In addition further work is required to develop tools adapted for specific populations tested to ensure acceptability and cultural competence. Lastly tools that are developed should be able to be used to determine risk and/or be sensitive enough to measure changes resulting from interventions.
